# Light and flow regimes regulate the metabolism of rivers

**DOI:** 10.1073/pnas.2121976119

**Published:** 2022-02-14

**Authors:** Emily S. Bernhardt, Phil Savoy, Michael J. Vlah, Alison P. Appling, Lauren E. Koenig, Robert O. Hall, Maite Arroita, Joanna R. Blaszczak, Alice M. Carter, Matt Cohen, Judson W. Harvey, James B. Heffernan, Ashley M. Helton, Jacob D. Hosen, Lily Kirk, William H. McDowell, Emily H. Stanley, Charles B. Yackulic, Nancy B. Grimm

**Affiliations:** ^a^Department of Biology, Duke University, Durham, NC 27708;; ^b^US Geological Survey (contractor), Water Mission Area, Reston, VA 20192;; ^c^US Geological Survey, Water Mission Area, Reston, VA 20192;; ^d^Department of Natural Resources and the Environment, University of Connecticut, Storrs, CT 06232;; ^e^Flathead Lake Biological Station, University of Montana, Polson, MT 59860;; ^f^Department of Plant Biology and Ecology, University of the Basque Country, Bilbao 48080, Spain;; ^g^Natural Resources and Environmental Science Department, University of Nevada, Reno, NV 89557;; ^h^School of Forest, Fisheries and Geomatics Sciences, University of Florida, Gainesville, FL 32611;; ^i^Nicholas School of the Environment, Duke University, Durham, NC 27708;; ^j^The Center for Environmental Sciences and Engineering, University of Connecticut, Storrs, CT 06232;; ^k^Department of Forestry & Natural Resources, Purdue University, West Lafayette, IN 47906;; ^l^Department of Natural Resources and the Environment, University of New Hampshire, Durham, NH 03824;; ^m^Center for Limnology, University of Wisconsin, Madison, WI 53706;; ^n^US Geological Survey, Southwest Biological Science Center, Flagstaff, AZ 86001;; ^o^School of Life Sciences, Arizona State University, Tempe, AZ 85287

**Keywords:** river ecosystems, metabolism, flow regimes, light regimes

## Abstract

Mean annual temperature and mean annual precipitation drive much of the variation in productivity across Earth's terrestrial ecosystems but do not explain variation in gross primary productivity (GPP) or ecosystem respiration (ER) in flowing waters. We document substantial variation in the magnitude and seasonality of GPP and ER across 222 US rivers. In contrast to their terrestrial counterparts, most river ecosystems respire far more carbon than they fix and have less pronounced and consistent seasonality in their metabolic rates. We find that variation in annual solar energy inputs and stability of flows are the primary drivers of GPP and ER across rivers. A classification schema based on these drivers advances river science and informs management.

## Significance

This paper provides a comprehensive analysis of the annual patterns of ecosystem productivity and respiration for more than 200 rivers, comparing the magnitude and phenology of river metabolic regimes with annual estimates from more than 150 terrestrial ecosystems. Although mean annual temperature and mean annual precipitation explain much of the variation in terrestrial productivity and are used to define biomes, for rivers the most important controls are annual light availability and flow stability. Attention to these gradients will substantially improve our ability to scale and model river ecosystem dynamics and may fundamentally change the way rivers are studied.

Examination of any list or map of the world’s biomes shows that freshwater ecosystems are either missing or are lumped into a single category for which a single estimate of productivity and net ecosystem carbon storage may be provided. In contrast, terrestrial ecosystems are subdivided into categories, often biomes, for which estimates of primary production are presented as a function of mean annual temperature and mean annual precipitation (1, 2). Identifying the biome in which any individual terrestrial research site is situated provides an easy mechanism for making initial assumptions about both the magnitude and phenology of ecosystem productivity and the potential for carbon storage. In contrast, knowing that a research site is within the “freshwater” category provides no such context. The absence of such basic categorical distinctions for rivers currently constrains the interpretation and synthesis across place-based aquatic ecosystem studies and amplifies the uncertainties associated with regional and global upscaling. For example, recent global estimates suggest that inland waters collectively retain or degas >50% of the reactive nitrogen and fixed carbon (C) they receive from their watersheds (3–6), but these extrapolations provide no information about where and when these cumulative global fluxes are being generated or how sensitive these processes may be to climate or land use change.

Techniques for measuring ecosystem energetics at daily time steps were pioneered in rivers (7, 8) before being applied to terrestrial ecosystems (9, 10), but aquatic sensor technologies have lagged terrestrially oriented technologies, such as eddy covariance towers and satellite remote sensing (10). The lack of rugged and durable sensors has stalled progress in river ecosystem energetics, with researchers limiting metabolism measurements to brief, supervised field campaigns under stable flow conditions. Such limitations constrain all modern attempts to synthesize and scale published estimates of annual or global river productivity and respiration (4, 11, 12) and to understand how widespread land use and climate change are altering river ecosystem energetics and thus, freshwater and terrestrial food webs (13, 14).

Recent advances in sensor technology that enable continuous measurement of dissolved oxygen concentrations (15) and modeling approaches that attribute that variation to photosynthesis, aerobic respiration, or atmospheric exchange (16) have made it possible to estimate annual rates of riverine gross primary production (GPP) and ecosystem respiration (ER) (17). Until recently, multiyear time series of riverine GPP and ER were limited to only a few rivers (18, 19); they are now available for hundreds (20). As these records accumulate, we can now document alternative phenologies of river ecosystems (21), scale ecosystem energy dynamics through fluvial networks (22–24), and link changes in river metabolic regimes to interannual climate variability (25, 26) and land use change (27, 28).

The goals of our collaborative effort, StreamPULSE, are to compare river and terrestrial ecosystem metabolism and estimate the factors that shape the seasonality and magnitude of river metabolism (17). We originally hypothesized that the primary drivers of variation in river metabolism would include their hydrologic regime, light regimes, and the magnitude and timing of terrestrial carbon inputs (17). We estimated rates of GPP, ER, and net ecosystem production (NEP) for 222 rivers within the United States for which we had near-continuous records of dissolved oxygen concentration for at least 1 full year (hereafter we will refer to these sites as StreamPULSE sites) (*SI Appendix*, Fig. S1) (29). Rates were estimated at a daily time step using the streamMetabolizer R package (16), allowing summation of annual fluxes and examination of seasonal patterns. We used a respiratory quotient of 1 mol C to 1 mol O to convert these oxygen-based estimates to carbon equivalents. We compared our compiled estimates of annual river ecosystem GPP, ER, and NEP with the range of values reported for 162 terrestrial ecosystems included in FLUXNET, a coordinated network of eddy flux towers and eddy covariance from which continuous rates of GPP and ER are estimates for terrestrial ecosystems across the globe (9) (site map is in *SI Appendix*, Fig. S1). We then developed a causal model of river GPP and ER as a function of river size, adjacent terrestrial ecosystem net primary production (NPP), solar energy inputs, and hydrology.

**Fig. 1.**
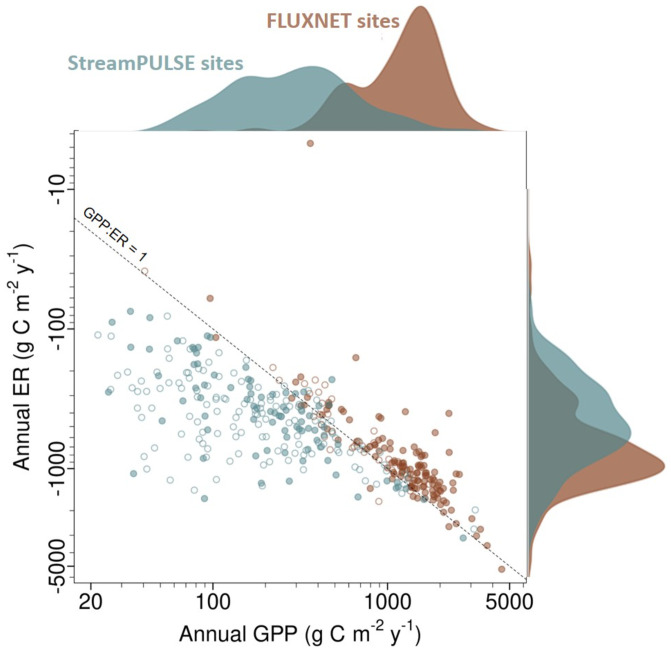
Annual rates of GPP and ER for 222 river and 162 terrestrial ecosystems are shown as a scatterplot relative to the 1:1 line of balanced aerobic ecosystem carbon production and consumption. The frequency distribution of GPP and ER values within each dataset is shown above and to the right of the scatterplot, with values aligned to the corresponding axis. Open circles indicate sites with at least 60% of all days in each year having estimated rates, and solid circles indicate sites with at least 80% of all days in each year having estimated rates. We show average annual values for sites with multiple years.

We found that river ecosystems range widely in rates of annual GPP and even more widely in rates of annual ER (Fig. 1). While a few rivers support remarkably high rates of carbon fixation and respiration (∼1,000 g C m^2^ y^−1^), most rivers have substantially lower annual ecosystem carbon fluxes than their terrestrial counterparts (Fig. 1). In contrast to the terrestrial ecosystems in the FLUXNET dataset, most of which accumulate carbon each year, the vast majority of river ecosystems are heterotrophic, with the median river respiring ∼200 g m^−2^ more carbon annually than is fixed through photosynthesis. We measured positive NEP for only 16 of 222 rivers in our dataset. For the rest, ER in excess of GPP must be sustained by the consumption and mineralization of terrestrial organic matter subsidies. Negative riverine NEP represents a carbon loss term that is poorly represented or missing from most terrestrial ecosystem models (30).

**Fig. 2.**
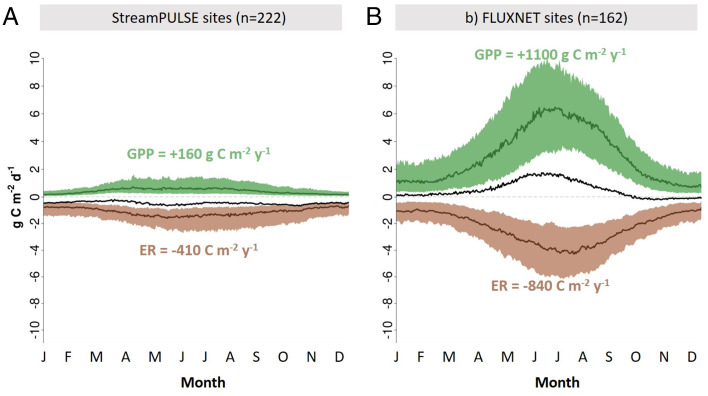
A comparison of river and terrestrial ecosystem metabolism. The median daily rates of GPP (green), ER (brown), and NEP (black) are shown as lines for 222 StreamPULSE rivers (*A*) and for 162 FLUXNET terrestrial ecosystems (*B*). The shaded area in each plot represents the interquartile range of values of GPP (green) and ER (brown) for each day. Median NEP is calculated as the difference between the two median values for each date and is the black line. For rivers with more than 1 site year, we used their average rate for each day of the year in this data synthesis. The estimated median values of annual GPP (green text) and ER (brown text) are estimated as the cumulative sum of the median daily flux.

The timing of peak annual GPP and ER varied across these 222 rivers, with at least some rivers having peak rates in nearly every month of the year (*SI Appendix*, Fig. S2) and many rivers having no annual peak (21). Many rivers have their highest GPP in the spring, while more than 20% of the rivers in our dataset have their highest ER in the autumn or winter. This substantial variation suggests that river ecosystems in the aggregate have weak metabolic seasonality and lack the distinctive “growing season” peak in GPP observed for most temperate terrestrial ecosystems (Fig. 2). NEP was persistently negative and relatively constant throughout the year for most StreamPULSE rivers, while most FLUXNET terrestrial sites accumulate carbon but only during summer (Fig. 2).

**Fig. 3.**
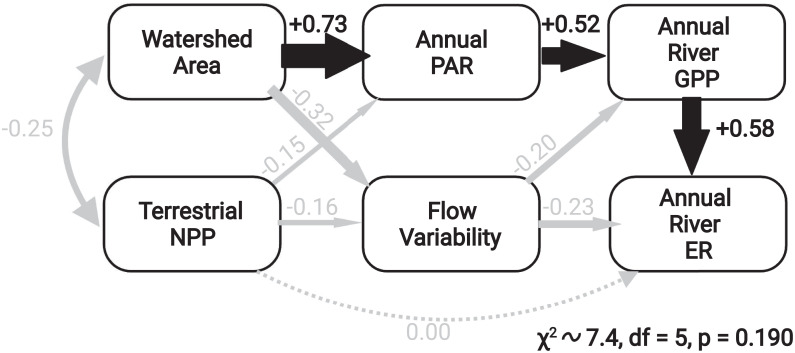
Structural equation model linking the watershed attributes (area, terrestrial NPP) and stream climate drivers (incoming photosynthetically active radiation [PAR] and flow variability) to GPP and ER across 222 rivers. The final model explained 35% of the variation in GPP and 47% of the variation in ER across sites. In this depiction, the size of the arrows is scaled to the standardized coefficients written alongside each arrow. Solid lines indicate statistically significant effects, while dashed lines indicate a hypothesized effect that was included in the initial model but for which there was no statistical support.

We are able to explain 35% of the variation in annual GPP and 47% of the variation in annual ER across the StreamPULSE dataset using a structural equation model (SEM) that includes 1) river size (a function of watershed area), 2) MODIS-based estimates of NPP for terrestrial vegetation surrounding each river segment, 3) availability of light to the channel surface (31), and 4) variability of streamflow measured as skewness (32) (Fig. 3). Annual GPP is highest where incident light is high and streamflows are steady. Variation in annual ER was primarily related to patterns of GPP, but more variable flow regimes also had direct negative effects (Fig. 3). Contrary to expectations, we found no support for a direct relationship between surrounding terrestrial NPP and river ER (Fig. 3), but we did observe that higher terrestrial NPP was correlated with more stable flows. Water temperature was initially included in our SEM but did not improve model fits.

**Fig. 4.**
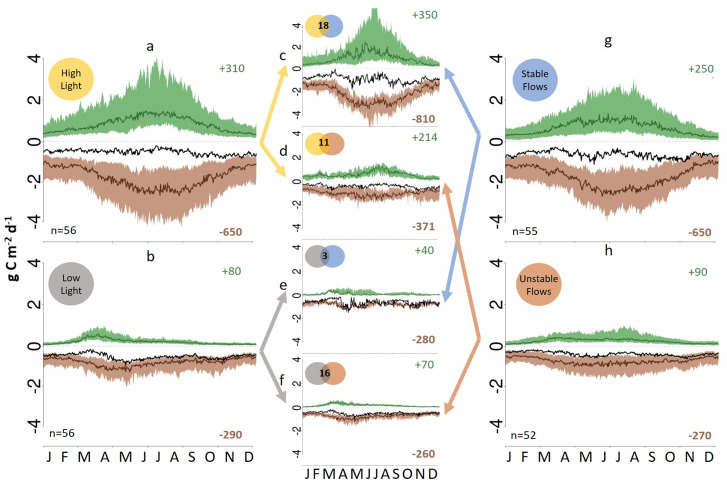
River metabolic regimes compared across light and flow regimes. *A* and *B* show the average seasonal patterns of GPP, ER, and NEP for sites in the highest quartile (*A*) and lowest quartile (*B*) of sites ranked by annual stream light. *G* and *H* show the same data for the highest quartile (*G*) and lowest quartile (*H*) of sites when ranked by flow variability (Q_skew). *C*–*F* show the average seasonal patterns of GPP, ER, and NEP for the joined subsets for sites that were in (*C*) the high-light, high-stability quartiles; (*D*) the high-light, unstable flows quartiles; (*E*) the low-light, stable flows quartile; and (*F*) the low-light, unstable flows quartiles. In each graph, the green number in the top right is the average annual GPP (grams C meter^−2^ year^−1^), and the brown number is the average annual ER (grams C meter^−2^ year^−1^) for each subsample. Larger versions of *C*–*F* are in *SI Appendix*, Figs. S3–S6.

Light energy is the primary limitation to GPP in many rivers (33). Stream surface light inputs are a function of latitude; channel width; topographic shading; and the stature, leaf area, and phenology of terrestrial vegetation along the channel. We estimated light available to each stream segment using the StreamLIGHT model (31), which calculates daily river surface light by accounting for the physical dimensions of the channel (width and azimuth) and time-varying light attenuation by terrestrial vegetation. Annual GPP was nearly four times higher for rivers in the most well-lit quartile (Fig. 4*A*) of our dataset than for rivers in the darkest quartile (Fig. 4*B*). In the low-light quartile, GPP peaks in early spring, while high-light rivers sustain productivity throughout the summer. These differences in river ecosystem phenology indicate that canopy shading strongly constrains summer GPP and thus, overall annual productivity in many rivers. Despite higher GPP, NEP was substantially more negative in the most well-lit rivers due to high rates of ER, which must be supported by organic matter inputs from upstream and riparian sources. We hypothesize that high light and an abundance of labile photosynthates in these well-lit rivers may also enhance rates of organic matter (OM) degradation by photolysis (34) and the cometabolism of more recalcitrant organic matter enabled by the availability of labile exudates (35).

The dominant autotrophs in freshwater ecosystems are benthic and planktonic algae and more rarely, rooted macrophytes, all of which have short life spans and stature compared with terrestrial plants (7). River autotrophs are frequently displaced, scoured, or buried by floods and desiccated during dry periods; thus, autotrophic biomass turns over quickly and may be removed many times annually in rivers with highly variable flow regimes (27, 36). Similarly, frequent bed-mobilizing flows are likely to constrain retention of organic matter and reduce both terrestrial and aquatic carbon contributions to ER. We thus expected that river flow regimes (37) would drive variation in metabolic rates across rivers. We compared the variability of flow regimes among sites by calculating daily flow distribution skewness based on L moments for each stream's annual hydrograph (32). For the most stable flow regimes (quartile with the lowest skew) (Fig. 4*G*), median annual GPP was three times higher than for the most variable flow regimes (highest skew) (Fig. 4*H*). Rivers with more variable flow regimes also have substantially lower ER (Fig. 4*H*) than more stable rivers. The magnitude of this hydrologic disturbance effect was substantially larger for ER than for GPP. As a result, rivers with more stable flow regimes produce more internal CO_2_ on an annual basis than those with highly variable flow regimes.

The absence of a direct positive relationship between riparian NPP and river ER (Fig. 3) implies a decoupling of terrestrial NPP and riverine ER and suggests that the capacity to store terrestrial OM within the river channel may be more important than OM loading in controlling ER. We hypothesize that the ability of rivers to consume and respire terrestrial OM inputs is highly contingent upon the mean residence time of fixed OM. During floods, rivers transport stored organic matter downstream and to adjacent floodplains, greatly reducing processing efficiency (38). Rivers with higher flood frequency, therefore, have shorter mean OM residence time, potentially decoupling riverine respiration from the terrestrial OM supply rate.

In contrast to terrestrial ecosystems (39), mean annual water temperature did not explain variation in rates of annual GPP or ER across rivers. In rivers, peak annual energy inputs of sunlight or allochthonous OM likely exert a larger effect than temperature and are often out of phase with thermal variation. While rising global water temperatures are likely to enhance both GPP and ER in rivers (38, 40), our results suggest that energy supply (as light or OM) and hydrologic disturbances will constrain where, when, and to what extent this amplification will occur.

**Fig. 5.**
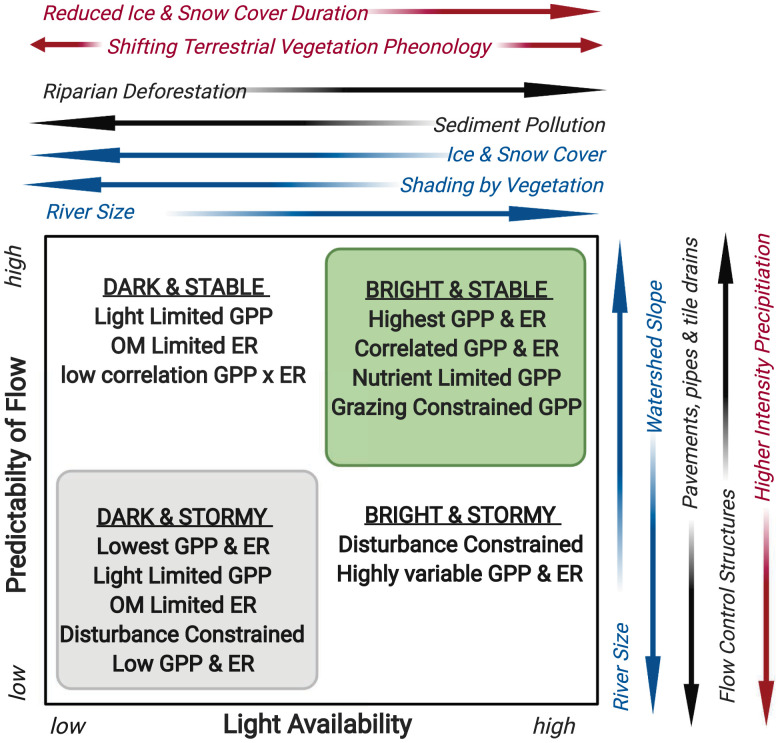
Our emerging conceptual understanding of ecosystem metabolism in rivers where productivity is often limited by light and constrained by physical disturbance. The light and flow regimes of rivers vary based on watershed topography, riparian vegetation, and river size (blue arrows), but these relationships may be regularly overwhelmed or superseded by management activities (black arrows) or changes in climate (red arrows) that alter flow and light availability. The four end-member descriptions provided here are well matched to the metabolic regimes depicted in Fig. 3.

We were unable to evaluate the effects of nutrient availability on river ecosystem metabolism because nitrogen and phosphorus data were either unavailable or derived from inconsistent methods for most StreamPULSE rivers. Unlike their terrestrial and lentic counterparts, nutrient limitation of autotrophs in rivers is less commonly observed (41, 42). We hypothesize that nutrients are most likely to constrain metabolic rates in rivers during periods of high light and stable flow (Fig. 5). Cross-site comparisons in rivers have found that both GPP and ER are strongly positively correlated with rates of nutrient uptake and negatively correlated with nutrient concentrations (43, 44). Further research is needed to distinguish between conditions in which nutrient supply limits river metabolism versus those where river metabolism regulates nutrient concentration and export.

Results from this synthesis of the annual metabolic regimes of 222 rivers underscore how landscape attributes profoundly influence processes in flowing waters (45) and demonstrate that flow and light regimes are the primary controls on the timing and magnitude of river ecosystem GPP and ER. We suggest that these two variables provide predictive power for classifying and upscaling riverine metabolism, analogous to mean annual temperature and mean annual precipitation for predicting variation in terrestrial productivity (1, 39). We expect that ordinating rivers along these two gradients (Fig. 5) will improve our estimates of the magnitude and seasonality of GPP and ER; our understanding spatial patterns in the distribution of life history traits and body forms of river animals; and our ability to predict how climate change, land use change, and water management alter metabolic rates and dominant organisms in affected rivers. River productivity and respiration are highest where light and thermal regimes coincide and where physical disturbances are infrequent. In contrast to lakes and coastal oceans where nuisance algal blooms are a primary concern of land use change, the trajectories for rivers in the Anthropocene are more likely to depend on changing light and flow regimes. Well-lit rivers with stable flows, including many regulated rivers, are susceptible to eutrophication and hypoxia as a result of nutrient enrichment and rising temperatures. However, for many rivers, the increased frequency of flooding and drying disturbances caused by climate, land use change, and water extraction may limit accumulation of autotrophic biomass and storage of organic matter in ways that reduce the availability and predictability of energy flow to support river food webs.

## Supplementary Material

Supplementary File

## Data Availability

All of the data and data analysis code used in the preparation of this manuscript are publicly available in an open source GitHub repository, https://github.com/streampulse/metabolism_synthesis, as well as on Figshare, https://doi.org/10.6084/m9.figshare.c.5812160.v3 (29). The raw sensor data from which stream metabolism estimates reported here are derived is also publicly available for download and visualization through our open science data platform https://data.streampulse.org/download_bulk.
