# Supplementary material for: Light and flow regimes regulate the metabolism of rivers
Source: Proc Natl Acad Sci U S A. 2022 Feb 14;119(8):e2121976119. doi: 10.1073/pnas.2121976119 (PMC8872740; doi:10.1073/pnas.2121976119)
Supplement: Supplementary File [file pnas.2121976119.sapp.pdf]

Supplementary Information for

Light and Flow Regimes Regulate the Metabolism of Rivers

Emily S. Bernhardt*[1†], Phil Savoy[1,2†], Michael J. Vlah[1], Alison P., Appling[2], Lauren E. Koenig[3,4], Robert O. Hall[4], Jr., Maite Arroita[5], Joanna R. Blaszczak[4,6], Alice M. Carter[1,4], Matt Cohen[7], Judson W. Harvey[2], James B. Heffernan[8], Ashley M.Helton[3], Jacob D. Hosen[9], Lily Kirk[7], William H. McDowell[10], Emily H. Stanley[11], Charles B. Yackulic[2], and Nancy B. Grimm*[13]*Corresponding Authors. Email: emily.bernhardt@duke.edu and nbgrimm@asu.edu

This PDF file includes:

Supplementary text
Figures S1 to S6
References

Supplementary Information Text

Materials and Methods

Lotic Metabolism data: Mean daily discharge ($m^3 s^{-1}$) and daily estimates of metabolism for lotic sites were compiled from (*1*) and the StreamPULSE data portal (https://data.streampulse.org). In both datasets, estimates of gross primary production (GPP) and ecosystem respiration (ER) were produced using the streamMetabolizer R package (*2*), which uses inverse modeling based on sub-daily time series of dissolved oxygen (DO), water temperature, discharge, depth, and photosynthetically active radiation (PAR). We limited our analysis to subset these datasets to only include sites within the continental United States so they could be linked to other nationally-consistent datasets. Biologically impossible values, negative GPP or positive ER, were excluded from the dataset and then individual site-years were filtered to only include years where the model generated reliable metabolism estimates for more than **60%** of all days of the year. Gaps in discharge or metabolism data were filled using a structural timeseries in the R statistical language (*3*). To facilitate comparison to terrestrial data, estimates of GPP and ER from lotic sites were converted from g O_2 $m^{-2} d^{-1}$ to g C $m^{-2} d^{-1}$ using a photosynthetic quotient of 1.25, which assumes a molar C:O ratio of 1:1.

FLUXNET : We used the FLUXNET2015 dataset (*4*) for terrestrial GPP and ER data. The FLUXNET2015 dataset contains data at multiple temporal resolutions and we used the annual sums of GPP and ER (g C $m^{-2} y^{-1}$) which were included in this dataset. However, only site-years with >60% of days having estimated rates were used in our analysis to be consistent with our approach to filtering lotic sites.

Stream Light estimates: Estimates of photosynthetically active radiation (PAR) at the stream surface were generated using the model of (*5*), which was implemented in the StreamLight R package (*6*). Specifically, we used the stream_light() function, which effectively assumes constant bankfull conditions for the purposes of determining wetted channel widths. Time series of incoming shortwave radiation (W m^{-2}) (*7*) and leaf area index ($m^2 m^{-2}$) (MCD15A2Hv006) (*8*) were used as inputs to the model in addition to several channel characteristics described below. The hourly estimates of PAR (µmol $m^{-2} s^{-1}$) were aggregated to daily sums (mol $m^{-2} d^{-1}$) so they would match the daily resolution of the stream metabolism estimates.

MODIS NPP data: For each of the lotic site-years in our final dataset, annual MODIS net primary production (NPP) data (kgC $m^{-2} y^{-1}$) (MOD17A3HGFv006)(*9*) was downloaded based on the site location through the Application for Extracting and Exploring Analysis Ready Samples (AρρEEARS) (*10*). NPP data were not available for all sites through AρρEEARS, possibly due to the proximity of pixels to MODIS water masks, so NPP data for the remaining sites were downloaded for a 1km buffer around the site location using the MODISTools package (*11*). For these sites, the mean value for all pixels in the buffer was calculated for each site-year of NPP. Finally, NPP was converted to g C $m^{-2} y^{-1}$ to be consistent with the other data sources used.

StreamPULSE Channel Characterization: Site locations were joined to their nearest flowline reach within the National Hydrography Dataset (*12*) using the nhdplusTools R package (*13*). From the paired flowlines, we assigned the Strahler stream order for each site. We also used the unique identification number for each flowline reach (i.e., the COMID) to gather relevant site information from additional linked datasets. Channel widths were estimated for each site location using either field-based measurements or geomorphic scaling relationships between width and cumulative upstream area. For the majority of sites, channel width was approximated as the median of width measurements available during the period of record for the metabolism estimates, acquired either from NWIS for sites co-located at USGS gages, or from the StreamPULSE data portal. For sites with no available width records, we estimated channel width based on empirical equations relating upstream area with width, regionalized at the HUC2 scale(*14*). Upstream watershed area was acquired from either the NHDPlus vaa tables based on the associated COMID. We calculated channel azimuths using the NHDPlusV2.1 flowlines, where the azimuth at each site was calculated as the circular mean of all segments within the associated reach. Tree heights were derived using 30m resolution global canopy height estimates from (*15*). Heights were extracted using the site coordinates and extending a 60m buffer into the riparian zone. The 90th percentile of canopy heights within the buffer for each site was returned. The 90th percentile of heights was chosen to capture tall canopy elements that could shade the channel.

StreamPULSE Hydrologic Regimes: The skewness in daily average discharge was calculated following (*16*). Specifically, skewness refers to L-skew. L-moments have been found to be useful for analyzing streamflow since they are relatively robust to outliers when compared to product moment estimators(*17*).

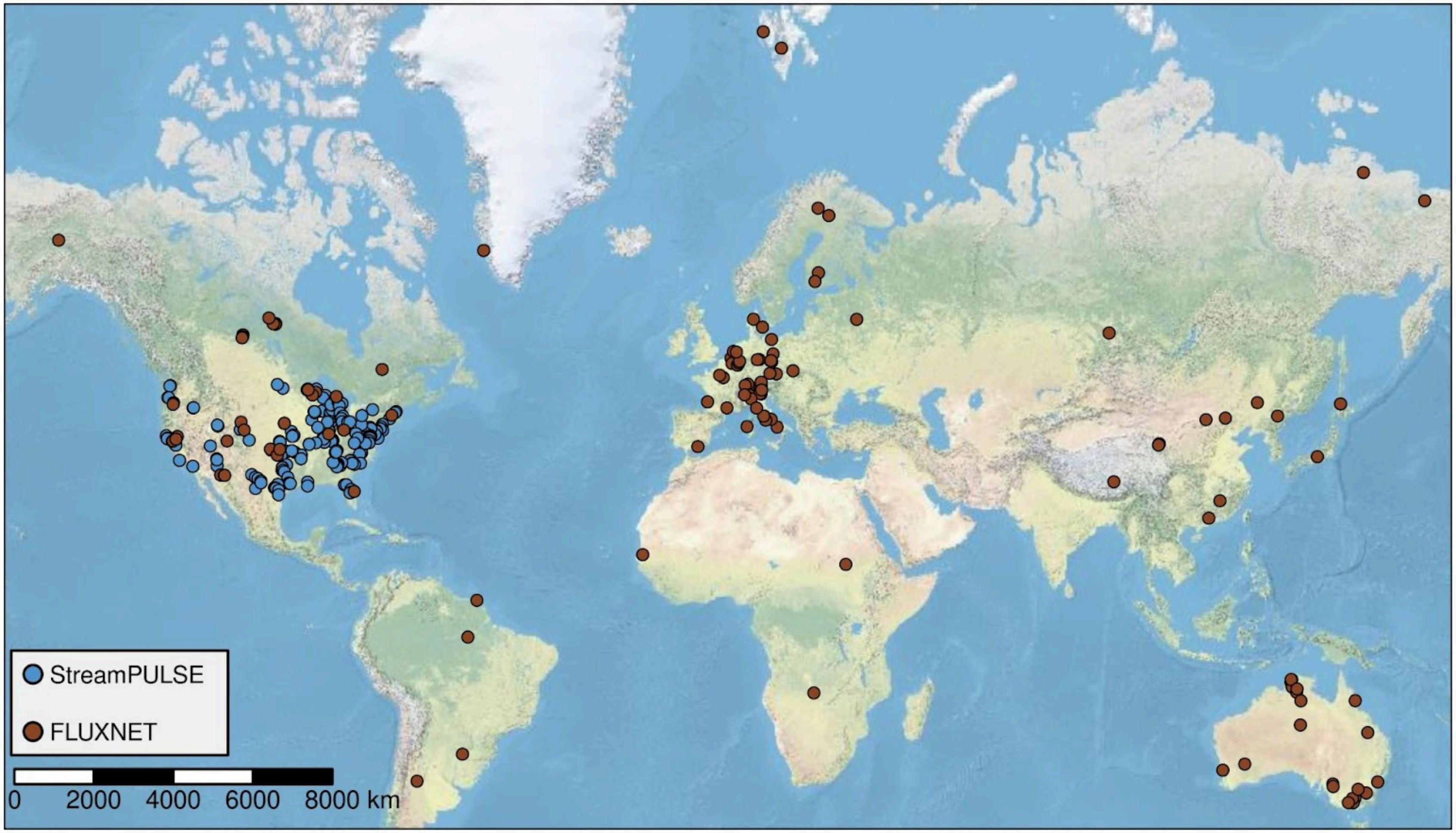

Figure S1. A map of the StreamPULSE and FLUXNET sites included in this analysis.

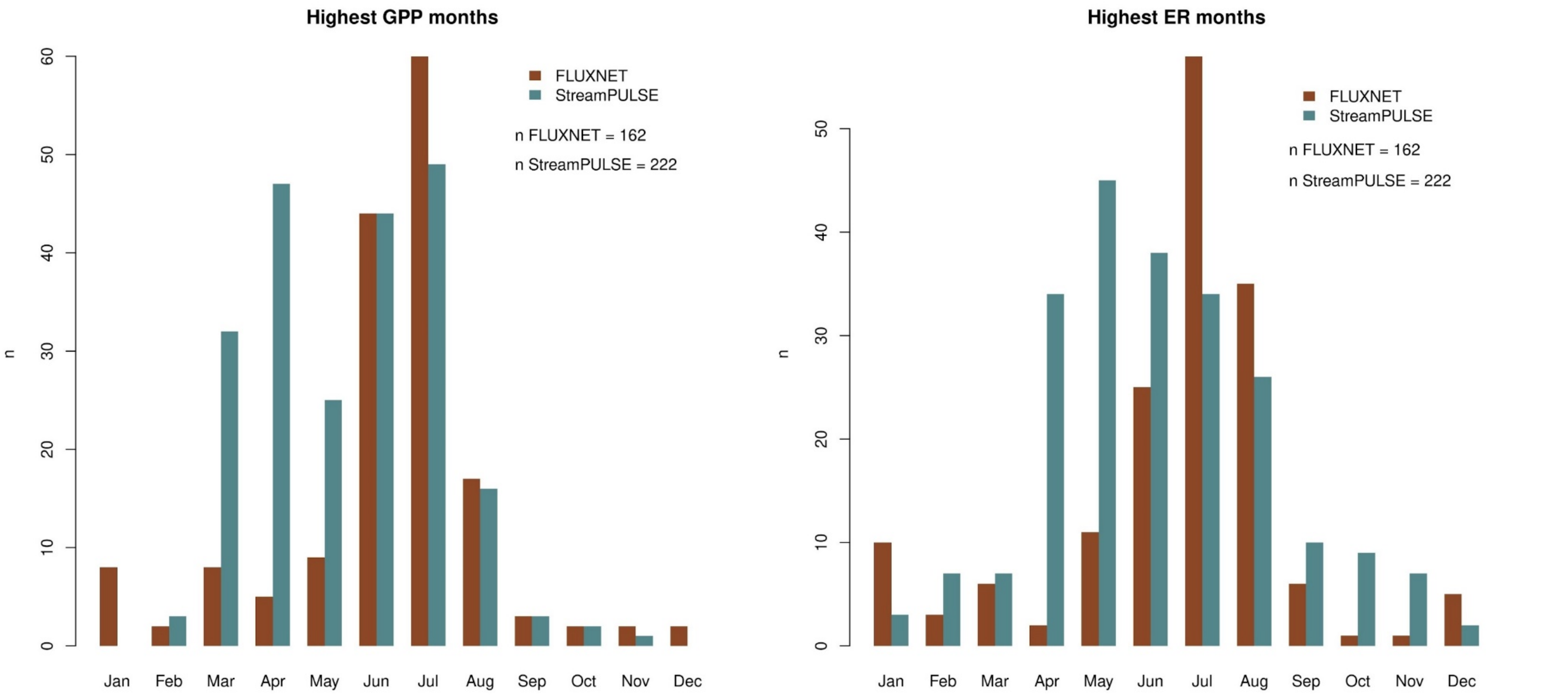

Figure S2: The number of StreamPULSE and FLUXnet sites with their peak daily rate of GPP (Panel A) and ER (Panel B) in each month of the year. Each site appears only once in each graph.

5

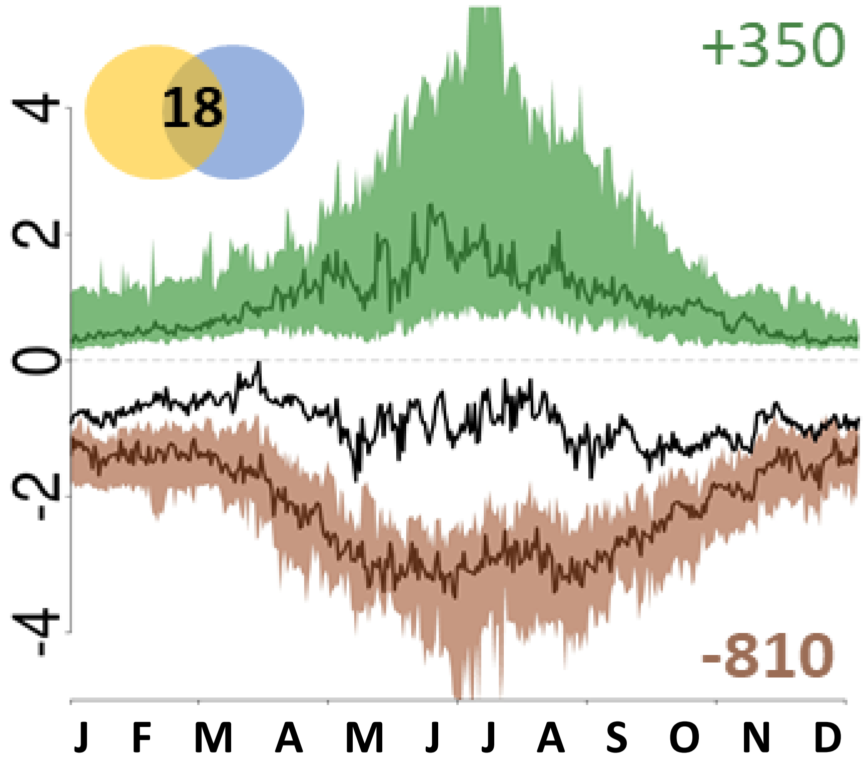

Figure S3: Metabolic profiles for the subset of BRIGHT (upper quartile of annual light) and STABLE (lower quartile of Q_skew) rivers in the StreamPULSE dataset (from Figure 4c).

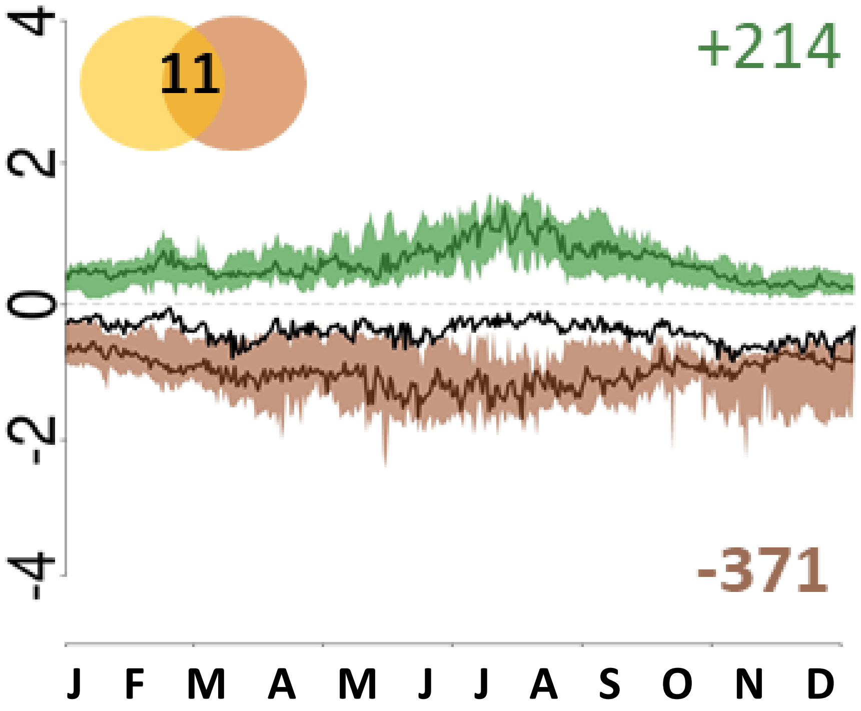

Figure S4: Metabolic profiles for the subset of BRIGHT (upper quartile of annual light) and STORMY (upper quartile of Q_skew) rivers in the StreamPULSE dataset (from Figure 4d).

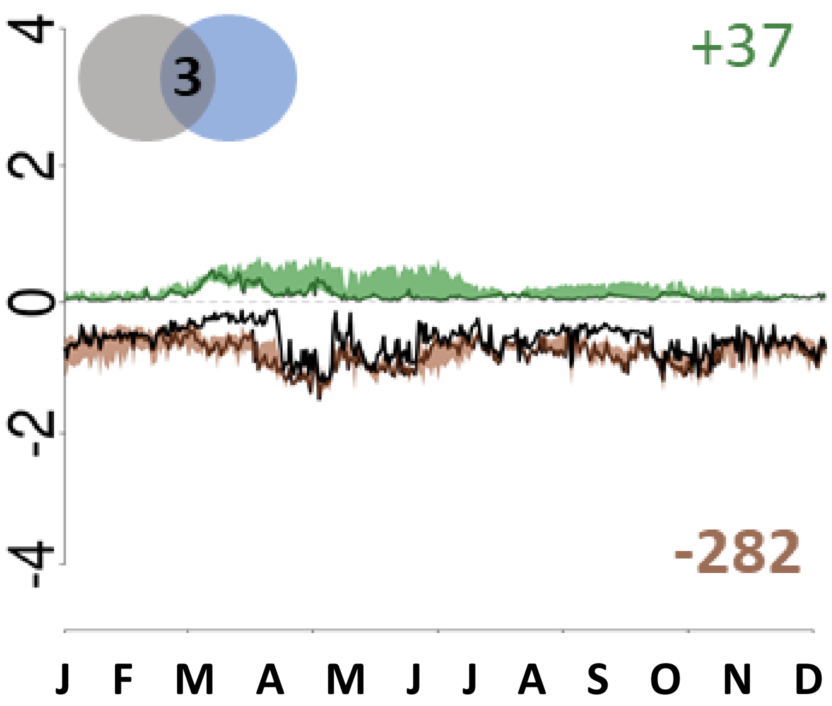

Figure S5: Metabolic profiles for the subset of DARK (lower quartile of annual light) and STABLE (lower quartile of Q_skew) rivers in the StreamPULSE dataset (from Figure 4e).

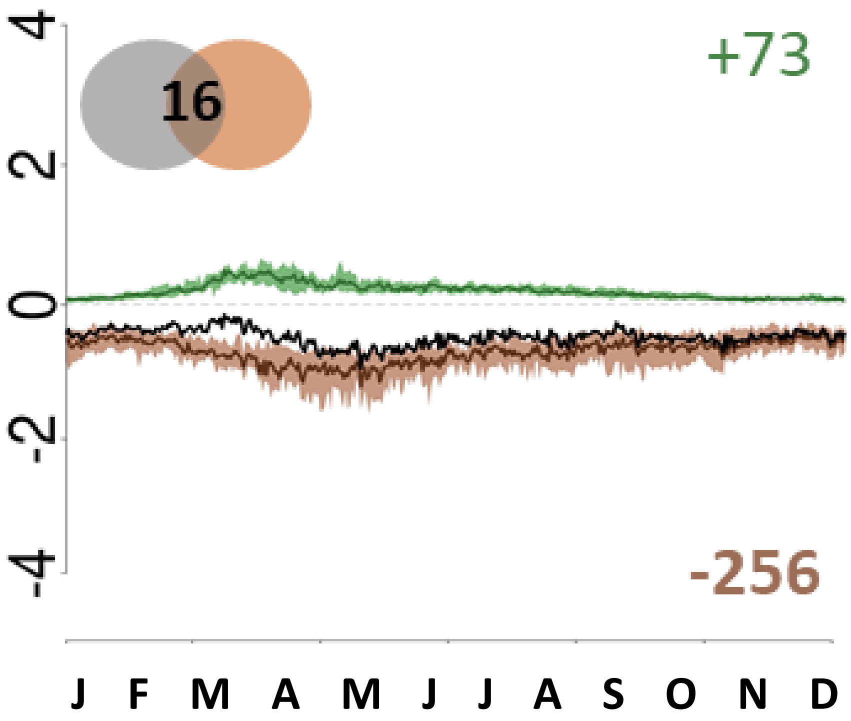

Figure S6: Metabolic profiles for the subset of DARK (lower quartile of annual light) and STORMY (upper quartile of Q_skew) rivers in the StreamPULSE dataset (from Figure 4f).

References

1. A. P. Appling, J. S. Read, L. A. Winslow, M. Arroita, E. S. Bernhardt, N. A. Griffiths, R. O. Hall Jr, J. W. Harvey, J. B. Heffernan, E. H. Stanley, E. G. Stets, C. B. Yackulic, The metabolic regimes of 356 rivers in the United States. *Sci Data*. **5**, 180292 (2018).

2. A. P. Appling, R. O. Hall Jr., C. B. Yackulic, M. Arroita, Overcoming equifinality: Leveraging long time series for stream metabolism estimation. *J. Geophys. Res. Biogeosci.* **123**, 624–645 (2018).

3. R Core Team, *R: A Language and Environment for Statistical Computing* (2019; https://www.r-project.org/).

4. G. Pastorello, C. Trotta, E. Canfora, H. Chu, D. Christianson, Y. W. Cheah, C. Poindexter, J. Chen, A. Elbashandy, M. Humphrey, Others, The FLUXNET2015 dataset and the ONEFlux processing pipeline for eddy covariance data, Sci. Data, 7, 225 (2020).

5. P. Savoy, E. Bernhardt, L. Kirk, M. J. Cohen, J. B. Heffernan, A seasonally dynamic model of light at the stream surface. *Freshw. Sci.*, **40** (2021) pp. 286-301 (2021).

6. P. Savoy, *StreamLight: An R package for estimating stream lighting conditions* (2021; https://github.com/psavoy/StreamLight).

7. Y. Xia, K. Mitchell, M. Ek, J. Sheffield, B. Cosgrove, E. Wood, L. Luo, C. Alonge, H. Wei, J. Meng, B. Livneh, D. Lettenmaier, V. Koren, Q. Duan, K. Mo, Y. Fan, D. Mocko, Continental-scale water and energy flux analysis and validation for the North American Land Data Assimilation System project phase 2 (NLDAS-2): 1. Intercomparison and application of model products. *J. Geophys. Res.* **117** (2012), doi:10.1029/2011jd016048.

8. Myneni, R., Knyazikhin, Y., Park, T., MCD15A2H MODIS/Terra+Aqua Leaf Area Index/FPAR 8-day L4 Global 500m SIN Grid V006 [Data set]. NASA EOSDIS Land Processes DAAC (2015), doi:10.5067/MODIS/MCD15A2H.006.

9. Running, S., Zhao, M. (2019). *MOD17A3HGF MODIS/Terra Net Primary Production Gap-Filled Yearly L4 Global 500 m SIN Grid V006* [Data set]. NASA EOSDIS Land Processes DAAC. Accessed 2021-09-08 from doi:10.5067/MODIS/MOD17A3HGF.006.

10. AppEEARS Team, *Application for Extracting and Exploring Analysis Ready Samples (AppEEARS)* (https://lpdaacsvc.cr.usgs.gov/appeears/`).

11. S. L. Tuck, H. R. P. Phillips, R. E. Hintzen, J. P. W. Scharlemann, A. Purvis, L. N. Hudson, MODIST ools – downloading and processing MODIS remotely sensed data in R. *Ecology and Evolution*. **4** (2014), pp. 4658–4668.

12. NHDPlusV2.1, (available at https://nhdplus.com/NHDPlus/NHDPlusV2_home.php).

13. D. Blodgett, *nhdplusTools: Tools for Using NHDPlus Data* (2019; https://github.com/dblodgett-usgs/nhdplusTools).

14. J. D. Gomez-Velez, J. Harvey, M. B. Cardenas, B. Kiel, Denitrification in the Mississippi River network controlled by flow through river bedforms. *Nat. Geosci.* **8**, 941–U75 (2015).

15. P. Potapov, X. Li, A. Hernandez-Serna, A. Tyukavina, M. C. Hansen, A. Kommareddy, A. Pickens, S. Turubanova, H. Tang, C. E. Silva, J. Armston, R. Dubayah, J. B. Blair, M. Hofton, Mapping global forest canopy height through integration of GEDI and Landsat data. *Remote Sens. Environ.* **253**, 112165 (2021).

16. S. A. Archfield, J. G. Kennen, D. M. Carlisle, D. M. Wolock, An objective and parsimonious approach for classifying natural flow regimes at a continental scale. *River Res. Appl.* **30**, 1166–1183 (2014).

17. J. R. M. Hosking, J. R. Wallis, *Regional Frequency Analysis: An Approach Based on L-Moments* (Cambridge University Press, 1997).
